# Long Shape Memory Alloy Tendon-based Soft Robotic Actuators and Implementation as a Soft Gripper

**DOI:** 10.1038/s41598-019-47794-1

**Published:** 2019-08-02

**Authors:** Ji-Hyeong Lee, Yoon Seop Chung, Hugo Rodrigue

**Affiliations:** School of Mechanical Engineering, Sungkyunkwan University, Suwon, 16419 Republic of Korea

**Keywords:** Electrical and electronic engineering, Mechanical engineering

## Abstract

Shape memory alloy (SMA) wire-based soft actuators have had their performance limited by the small stroke of the SMA wire embedded within the polymeric matrix. This intrinsically links the bending angle and bending force in a way that made SMA-based soft grippers have relatively poor performance versus other types of soft actuators. In this work, the use of free-sliding SMA wires as tendons for soft actuation is presented that enables large increases in the bending angle and bending force of the actuator by decoupling the length of the matrix and the length of the SMA wires while also allowing for the compact packaging of the driving SMA wires. Bending angles of 400° and tip forces of 0.89 N were achieved by the actuators in this work using a tendon length up to 350 mm. The tendons were integrated as a compact module using bearings that enables the actuator to easily be implemented in various soft gripper configurations. Three fingers were used either in an antagonistic configuration or in a triangular configuration and the gripper was shown to be capable of gripping a wide range of objects weighing up to 1.5 kg and was easily installed on a robotic arm. The maximum pulling force of the gripper was measured to be 30 N.

## Introduction

Soft robots present several advantages over their traditional counterparts in that they are compliant which allows them to interact adaptively and safely with their environment and with humans without requiring complex and costly control systems that can require sensors or large computational power^1–3^. Pneumatic and motor-driven tendon systems have, so far, been the most promising types of soft actuators for implementation in various robotic applications such as soft wearables or soft grippers^4–8^. A wide range of soft actuators and smart materials have been developed with diverse capabilities and diverse fabrication methods, but SMAs are silent, have a high power density and require only an electric input for actuation, but their performance is often lacking when implemented in a soft robotic system for real-life applications^9–12^. Various designs of soft actuator consisting of a Shape memory alloy (SMA) wires paired with a polymeric matrix have been proposed, but their design limitations severely limit their potential for real-life applications^13^.

SMA wires have a limited linear stroke in the range of 4 to 8% of their length^14,15^, but it is possible to transform this small deformation into large out-of-plane deformations by embedding them in a polymeric matrix^16–18^. Anisotropic elements can be added to the polymeric matrix to produce coupled bending and twisting deformations^19,20^, and multiple SMA wires can be used to produce twisting deformations or multiple types of deformations in a single actuator^21,22^. Complex SMA wire positioning can be obtained by double casting the actuator to produce a hollow-tube shaped actuator with multiple SMA wires positioned as helix to sustain lateral loads while producing a twisting deformation^23^. High current can be used to produce rapid movements of the actuator that can make robots jump^24^. These actuators can be used to produce crawling robots^25,26^, swimming robots^27–29^, blooming flowers^30^, or complex deployable structures^31,32^. Grippers where embedded rigid segments are embedded to produce jointed deformations have been developed^33^, grippers with varying thickness to create hinge-like motions have been developed^34^, and grippers where fusible alloy elements are used to selectively stiffen the fingers when grasping^35^. Curved SMA-based actuators have been used to produce larger maximum deformations, but their grasping force as a soft gripper remains insufficient for grasping most objects^36^. SMA wire-based soft actuators and grippers with embedded SMA elements have had the usable length of the SMA wire limited to the length of the matrix when embedding it within the matrix during the casting process, which produces a significant trade-off between force and deformation.

SMA springs have been used externally to the deforming structure to produce crawling robots using a peristaltic motion^37^, using origami-based folded structure^38^, robots capable of a bio-inspired rolling motion^39^, octopus tentacles^40^, jumping robots^41^, to cause the deformation of bistable structures^42,43^, and grippers using externally located SMA springs with free-sliding tendons to transmit their force^44^. SMA wires with torsional pre-strain have also been used to deform origami-based structures^45,46^. SMA plates have been used in a soft finger with significant force, but requires too much power for practical applications^47^. Twisted and coiled actuators (TCA) made from nylon fishing lines or other polymers can be used to produce large contraction with limited forces^48,49^, dielectric elastomers can produce rapid and small deformations^50^, ionic polymer-metal composites can produce diverse deformations through the movement of cations in a ionic membrane under an applied voltage^51^, and phase changing materials can expand significantly when heated^52^. All the surveyed smart material-based actuators are limited in some factors such as their force, usable strain, size limitations, implementation requirements or speed.

In this paper, we present the use of SMA wires themselves as free-sliding tendons to drive a polymeric matrix which allows the SMA length and the design of the polymeric matrix to be decoupled while also allowing the compact packaging of the driving SMA wires through mechanical systems. This allows greater control for designing actuators based on the required bending angle and force characteristics. The effect of the length of the tendon on the bending angle is presented and compared with a quasi-static numerical model, and the effect of the tendon length on the tip force is evaluated. This SMA wire tendon concept is then applied to a compact and modular module using pulleys to compactly package the SMA wire which was implemented in a soft gripper that can be mounted to any robotic arm and used to grasp a wide range of objects. The gripper was tested with a wide range of object shapes and weights, tested using a tensile testing machine, and mounted on a robotic arm.

## Methods

### Concept and manufacturing

**Figure 1:**
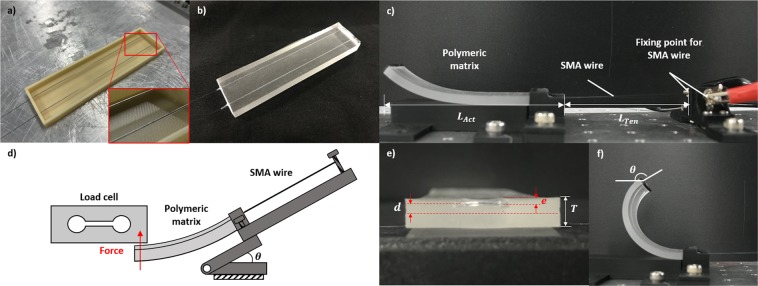
(**a**) Mold with the SMA wires positioned within silicone rubber tubes, (**b**) manufactured actuator, (**c**) fixing point and length dimensions of the actuator, (**d**) jig for measuring the tip force of the actuator, (**e**) cross-section of the actuator, and (**f**) bending deformation of the actuator.

The proposed actuator consists of a long SMA wire with a diameter of 203 µm (Flexinol LT, Dynalloy) passed through a shorter silicone rubber tube with an inner diameter of 300 µm (McMaster-Carr). The SMA wire is then positioned as a U-shape longitudinally in a rectangular 3D printed acrylonitrile butadiene styrene (ABS) mold with holes for positioning of the SMA wire. The silicone tube should cover the portion of the SMA wire that is within the mold matrix (Fig. 1a). Mixed and degassed polydimethylsiloxane (PDMS, Sylgard-184, Dow-Corning) is then poured into the mold and cured at 55 °C for 8 hours. The mold is then cut and discarded (Fig. 1b).

This concept allows the SMA wire to slide freely within the polymeric matrix through being positioned within a slightly larger and hollow channel in the matrix formed by the silicone tube. In previous research, the SMA wire was placed in the mold during curing of the polymer and is in direct contact with the matrix such that its effective length is limited to that of the matrix. Each end of the SMA wire are then fixed externally using a bolt at the desired length, and a current of 0.95 A is applied where the SMA wire is fixed (Fig. 1c). This current was chosen empirically as the actuator can reach its maximum deformation within 1 s while being capable of continuous actuation without overheating. This concept allows for the length of the SMA wires to be independent from that of the matrix, and thus the stroke length of the SMA wires depends on the recovery strain of the wire and its length rather than the matrix length. To measure the tip force, the actuator is fixed on a jig whose angle can be varied using a guide and where the tendon length can be adjusted, and a load cell is used to measure magnitude of the tip force (Fig. 1d)

### Quasi-static model

The model consists of finding the equilibrium point where either the stress required in the SMA wire to further increase the bending angle is equal to the stress required to bend the matrix. The force *F*_*SMA*_ of the SMA wire can be calculated based on the stress *σ*_*SMA*_ within the SMA wire and area *A*_*SMA*_ of SMA wire within the cross-section of the actuator as

1 $${F}_{SMA}={A}_{SMA}{\sigma }_{SMA}$$

The strain *ε*_*CP*_ of the center plane of the actuator and the strain *ε*_*AP*_ within the actuator in the plane the SMA wire can be calculated as

2 $${\varepsilon }_{CP}=\frac{{F}_{SMA}}{{E}_{Act}{A}_{Act}}$$

3 $${\varepsilon }_{AP}=\frac{{F}_{SMA}{d}^{2}}{{E}_{Act}{I}_{Act}}+\frac{{F}_{SMA}}{{E}_{Act}{A}_{Act}}$$

where *d* is the eccentricity of the SMA wire with the neutral plane (Fig. 1e), *E*_*Act*_ is the Young’s modulus of the actuator, *A*_*Act*_ is the area of the cross-section of the actuator, *d* is the eccentricity of the SMA wire with respect to the center plane, and *I*_*Act*_ is the moment of inertia of the rectangular actuator across its length. The bending angle *θ* of the actuator of the actuator can then be calculated based on the length *L*_*Act*_ of the actuator as

4 $$\theta =\frac{({\varepsilon }_{AP}-{\varepsilon }_{CP}){L}_{Act}}{d}$$

The actual strain *ε*_*SMA*_ of the SMA wire can then be calculated as

5 $${\varepsilon }_{SMA}=\frac{{\varepsilon }_{AP}{L}_{Act}}{{L}_{Act}+{L}_{Ten}}$$

Where *L*_*Ten*_ is the length of the tendon (Fig. 1f). If the length of the SMA wire is equal to that of the matrix, as in the surveyed literature, then *L*_*Ten*_ is equal to zero and could be used to solve simple bending actuators. The stress *σ* produced by the SMA wire can then be related to the strain and temperature within the wire using the Brinson thermoconstitutive model for one-dimensional SMA elements as follows^15^

6 $$\sigma -{\sigma }_{0}=E(\xi )\varepsilon -E({\xi }_{0}){\varepsilon }_{0}+\Omega (\xi )\xi -\Omega ({\xi }_{0}){\xi }_{0}+\Theta (T-{T}_{0})$$

Where *σ*_0_ is initial stresses in the wire assumed to be zero, *E* is the Young’s modulus of SMA which depends on the martensite fraction of the SMA, *ε* and *ε*_0_ are the current and initial strains in the SMA wire where we assume that *ε*_0_ = *ε*_*L*_, *Ω* is the phase transformation contribution equal to *−EεL*, *ε*_*L*_ is the maximum recoverable strain, *ξ* and *ξ*_0_ are the current and initial martensite fractions assuming that the martensite is fully detwinned during pre-straining of the SMA wire and also during cooling of the actuator due to the elastic force of the matrix such that *ξ*_0_ = 1, *Θ* is the thermoelastic coefficient assumed to be equal to 0, and *T* and *T*_0_ are the current and initial temperatures. Re-arranging and applying assumptions, the following equation is obtained in terms of the strain of the SMA wire

7 $$\varepsilon =\frac{\sigma }{\xi {E}_{m}+(1-\xi ){E}_{a}}+{\varepsilon }_{L}\xi $$

Where *E*_*a*_ is the Young’s modulus of SMA in the austenite phase, and *E*_*m*_ in the martensite phase. The martensite fraction *ξ* during strain recovery can be calculated as

8 $$\,\xi =\frac{{\xi }_{M}}{2}(\cos [\frac{\pi (T-{A}_{s})}{{A}_{f}-{A}_{s}}-\frac{\pi \sigma }{{C}_{A}({A}_{f}-{A}_{s})}]+1)$$

Where *ξ*_*M*_ is the minimum martensite fraction, which is assumed to be equal to 1 as actuation starts from the fully martensite state, *C*_*A*_ is a dimensionless curve-fitting parameter equal to 10.3 based on previous work on previous literature^53,54^, and *A*_*s*_ and *A*_*f*_ are the austenite start and finish transition temperatures. Equations 6, 7 and 8 are then used to calculate the stress produced in the SMA throughout the strain recovery, and equations 4 and 5 are used to calculate the stress and strains required by the SMA to deform the actuator such that the point where both intersect is the quasi-static equilibrium of the actuator.

This model can be used to predict the bending deformation of the actuator for a given SMA wire tendon length and polymeric matrix combination, which is useful in reducing testing time when developing soft robotic components using this actuator design.

## Results

### Bending angle experiments

The basic goal of using SMA tendons to actuate a polymeric matrix is to use an SMA wire longer than the length of the matrix to increase its performance. The performance factor that is most directly impacted by using a longer SMA wire is the bending angle of the actuator. It is expected that it is possible to obtain large increases in the bending angle of the actuator by using a longer SMA wire either up to the force limit of the SMA wire or up to the physical limit of the deformation. To demonstrate this, an experiment is conducted where three actuators with matrix dimensions of 100 mm in length and 25 mm in width are manufactured with thicknesses of 3, 5, and 8 mm. The length of the SMA wire embedded in the actuator is approximately one meter such that the maximum tendon length is 400 mm since it functions as two wires in parallel. However, the maximum usable length is 350 mm due to needing to fasten the SMA wire at each end. Each actuator is fastened at its base, and the fixation point of the SMA wire is moved incrementally from 0 to 350 mm or until the physical limit of the deformation of the actuator. A tendon length of 0 mm corresponds to that of an actuator with the embedded SMA wire not in tendon configuration, which is what is used in most previous SMA wire-based soft actuators.

**Figure 2:**
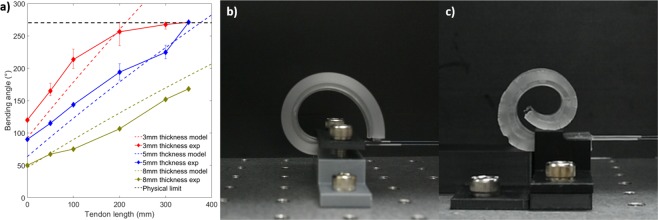
(**a**) Bending angle of actuator with different thicknesses for different tendon lengths, (**b**) maximum bending angle of the actuator, and (**c**) deformation of the actuator with a tapered length.

Results show that the actuator with a thickness of 3 mm produces a bending angle of 213° with a tendon length of 100 mm and reaches its physical bending limit at a bending angle 270° and a tendon length of 200 mm (Fig. 2a). This limit is due to the tip of the actuator touching the actuating SMA tendons such that further deformation is not possible (Fig. 2b). The bending angle of the actuator with a matrix thickness of 5 mm increases from 143° with a tendon length of 100 mm and increases linearly with an increase in tendon length until also reaching its physical bending limit at a tendon length of 350 mm. The actuator with a thickness of 8 mm produces a bending angle of 75° with a tendon length of 100 mm and its bending angle increases up to 168° with a tendon length of 350 mm. These results correspond well with the model up to the physical limit of the bending deformation. A large increase in bending angle can be seen versus the actuator with a tendon length of 0 mm for all thicknesses.

The tests for the actuators with a matrix thickness of 3 and 5 mm showed that the bending angle of the actuator may be limited by the actuator folding onto either itself, the jig or the driving SMA wires. Using the proposed tendon concept, it is possible to choose the desired matrix shape of the actuator such as to produce a desired deformation. A matrix with a tapered thickness from 5 mm at its base to 2.5 mm at its tip was used to produce a larger deformation at the tip such that the bending motion can avoid bending on itself. With a tendon length of 680 mm, the actuator can produce a bending angle of 400° (Fig. 2c), which is the largest bending angle of all SMA-based soft actuator reported in the surveyed literature.

### Bending force experiments

**Figure 3:**
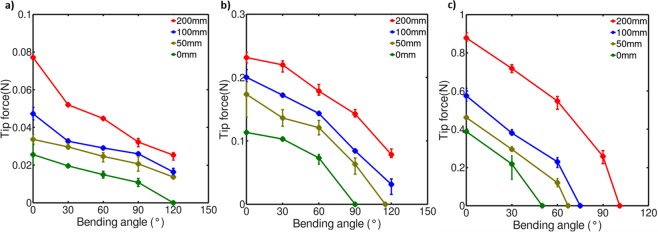
Tip force for different tendon lengths of actuators with a thickness of (**a**) 3 mm, (**b**) 5 mm, and (**c**) 8 mm.

Another important performance factor to assess soft bending actuators is the bending force of the actuator. In this work, this force was evaluated by fixing the actuator and measuring its tip force at different bending angles to obtain the force profile of the actuator. The tip force of the actuator was measured for actuators with thicknesses of 3, 5, and 8 mm with tendon lengths of 0, 50, 100 and 200 mm (Fig. 3a–c). The tip force was measured at bending angles in increments of 30° by using a load cell (CB1-600gf, Da Cell). The maximum measured angle of the force of the actuator was 120° due to not having sufficient space to place the load cell at bending angles above this value.

All four actuators produced their maximum tip force at the 0° position and continued to produce a bending force if the measured angle is smaller than the maximum bending angle for the tested actuator thickness and tendon length configuration. All actuators exhibited a nearly linear decrease in tip force throughout the deformation from the value measured at a 0° bending angle up to the maximum measured angle. Thicker actuators also produce a larger tip force throughout most of their deformation. This could be due to the thinner actuators not being able to transmit forces to the tip of the actuator as well as thicker ones due to deformations between the base and the tip of the actuator.

When the active length of the SMA wire is limited to that of the matrix, actuators making use of a thicker matrixes would produce smaller bending deformations which would reduce the usefulness of the actuator despite being capable of larger forces than thinner actuators. The use of long driving SMA wire tendons produces larger deformations and larger forces. It also becomes possible to retain the same bending deformation with a thicker matrix by using a longer tendon and thus scale up even further the force of the actuator.

### Behavior using uneven tendon length

**Figure 4:**
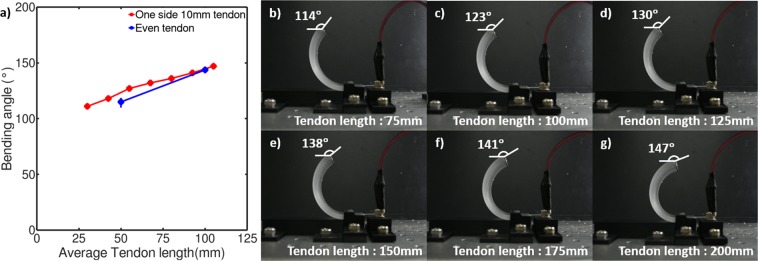
(**a**) Bending angle comparison for even tendon lengths and uneven tendon lengths, and bending deformation for actuators with one tendon length of 10 mm and second tendon length of (**b**) 75 mm, (**c**) 100 mm, (**d**) 125 mm, (**e**) 150 mm, (**f**) 175 mm, and (**g**) 200 mm.

The SMA tendons are able to slide freely within the actuator and the length of the tendon at each end can be adjusted such that each end has a different tendon length. The actuator with a matrix thickness of 5 mm was tested with one tendon having a fixed length of 10 mm and the other having its tendon length varied from 50 to 200 mm in 25 mm increment (Fig. 4a). It can be observed visually that the actuator produces a pure bending deformation with no torsion (Fig. 4b–g), which shows that the uneven tendon lengths do not cause one side of the actuator to contract more than the other. The bending deformation increases linearly with an increase in the tendon length and follows an increase similar to that of the actuator with even tendon lengths. These results show that the contraction of the tendon is distributed in each side due to its ability to slide freely within the silicone tube. This behavior may be helpful when grasping irregular or elliptical objects whose surface would require a slight rotation of the actuators.

### Robotic gripper

**Figure 5:**
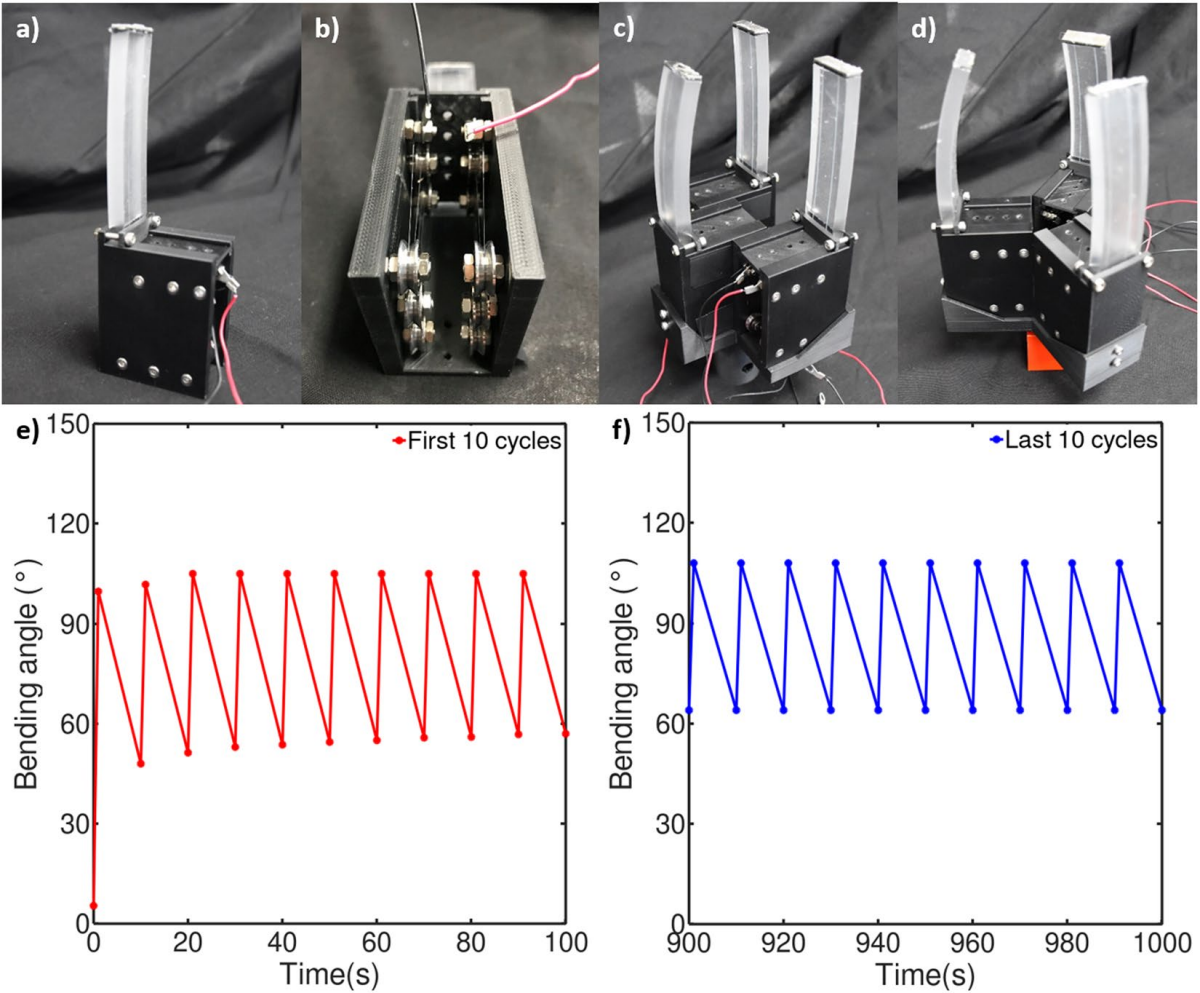
(**a**) Single actuator module, (**b**) inside view of the module, (**c**) gripper with antagonistic configuration, and (**d**) gripper with triangular configuration, (**e**) the first 10 cycles and (**f**) the last 10 cycles of a 100-cycle module actuation test.

A robotic gripper making use of long tendons and a thicker polymeric matrix should be able to have improved grasping performance versus previously developed SMA-based grippers due to the improved bending deformations and tip forces that were demonstrated in the previous section. However, the implementation of 400 mm long tendons requires a tendon routing solution to produce a compact gripper that can be implemented for diverse applications. In this work, each finger of the robotic gripper is built as a stand-alone module with a matrix thickness of 8 mm and a tendon length of 300 mm, whose tendon is routed through a series of V-shaped bearings (Fig. 5a,b). These modules can then be assembled modularly to produce a gripper of the desired shape and number of fingers. This modularity allows for ease of repair and customization. The basic configuration tested in this work is that of a three-fingered grasper with two of the fingers in parallel and the other placed antagonistically (Fig. 5c), and a triangular formation of three fingers was also tested (Fig. 5d). The module was tested for 100 cycles with 1 second of heating and 9 seconds of cooling time, and no degradation in the maximum bending angle of the actuator was visible (Fig. 5e,f). The minimum bending angle during cooling increased due to heat accumulation within the matrix, but the actuator returns near its original unactuated bending angle after being allowed to fully cool.

**Figure 6:**
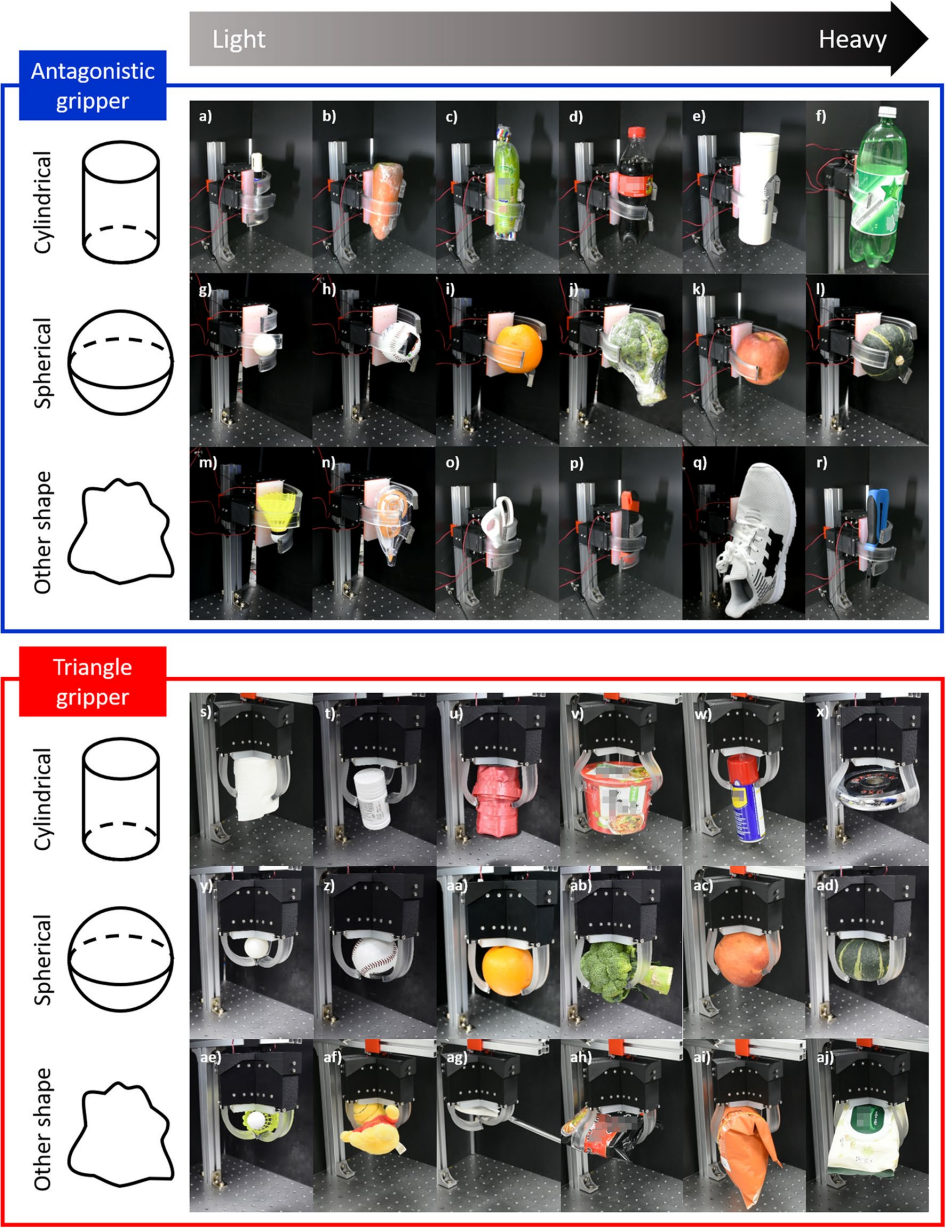
Antagonistic gripper in the horizontal position gripping (**a**) a marker(17 g), (**b**) a carrot(200 g), (**c**) a zucchini(250 g), (**d**) a bottle of soft drink(310 g), (**e**) a tumbler(263 g), (**f**) a large bottle of soft drink(1513 g), (**g**) a ping-pong ball(27 g), (**h**) a baseball(130 g), (**i**) an orange(240 g), (**j**) a broccoli(300 g), (**k**) an apple(305 g), (**l**) a sweet pumpkin(311 g), (**m**) a shuttlecock(5 g), (**n**) correction tape(45 g),(**o**) a pair of scissors(85 g), (**p**) a box cutter(128 g), (**q**) a shoe(180 g), and (**r**) a ratcheting crimper pliers(420 g). Triangular gripper in the vertical position gripping (**s**) toilet paper(86 g), (**t**) a bottle of medicine(95 g), (**u**) a roll of string(126 g), (**v**) a cup of noodles(150 g), (**w**) oil can(215 g), (**x**) a weight of 2 kg(2000g), (**y**) a ping-pong ball(27 g), (**z**) a baseball(130 g), (**aa**) an orange(240 g), (**ab**) a broccoli(300 g), (**ac**) an apple(305 g), (**ad**) a sweet pumpkin(311 g), (**ae**) a shuttlecock(5 g), (**af**) a stuffed toy(40 g), (ag) a pair of scissors(85 g), (ah) a pack of instant noodles(120 g), (ai) a bag of chips(135 g), and (**aj**) wet towel wipes(173 g).

The static adaptive grasping capability of the actuator was tested by grasping objects with a wide range of shapes and weights by applying a fixed current of 0.95A to the SMAs for all objects regardless of their shape or weight. When using adaptive soft grippers, the main goal is to be able to grab objects with different shapes, sizes and weights regardless of their exact position such that can be used in less structured environments. The antagonistic gripper was tested in the horizontal position as this position makes it harder to hold onto the object, and tested objects include a wide range of object shapes and sizes with cylindrical, spherical and other shapes with weights ranging from 5 to 1513 g (Fig. 6a–r). The heaviest and largest object held by the gripper is a full 1.5 L bottle of soda which was able to be grasped both in the vertical and horizontal position. The gripper with a triangular finger configuration is intended for use in the vertical position and was tested for a wide range of objects in the vertical position in which case both power grasps, when the inside of the actuators is holding the object, was realized for smaller objects and fingertip grasps, when the tip of the actuators is holding the object, was realized for larger objects (Fig. 6s–aj). The first gripper was particularly well suited for cylindrical-shaped objects while the second gripper was better suited for round objects. However, both configurations proved capable of grasping either object shapes.

**Figure 7:**
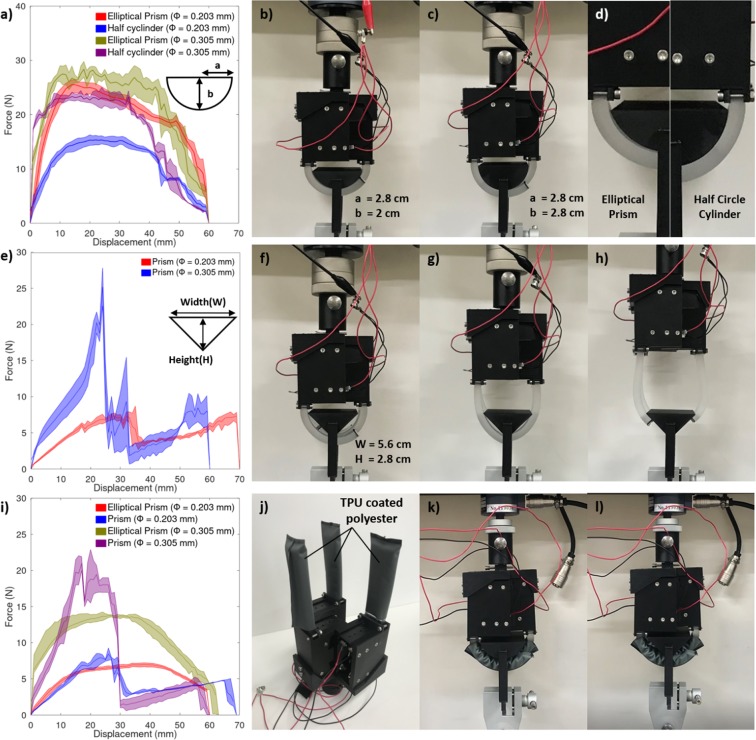
(**a**) pulling force of the gripper for an elliptical prism and a half cylinder, (**b**) gripping shape for the elliptical prism, (**c**) gripping shape for the half cylinder, (**d**) comparison of the two jigs, (**e**) pulling force for the inverted triangular prism, (**f**) initial position with the inverted triangular prism, (**g**) before transitioning to fingertip grasp, and (**h**) in fingertip grasp. (**i**) Pulling force with the fingers covered with a TPU coated polyester sleeve, (**j**) schematic of the gripper, (**k**) setup with the elliptical prism jig, and (**l**) setup with the inverted triangular prism jig.

When the gripper is grabbing an object using a power grasp, the grasping force of the gripper increases when a larger area of the actuators is in contact with the object. The pulling force of the gripper was evaluated for grippers with SMA wires with SMA wire diameters of 0.203 mm and 0.305 mm using a tensile testing machine (QC-506, Cometech) using three jigs where the first is an elliptical prism with radiuses of 28 and 20 mm, the second is a half cylinder with a radius of 28 mm, and the third is an inverted triangular prism with a width of 56 mm and a height of 28 mm (Fig. 7a–h). The elliptical prism was designed such that a significant portion of the jig is in contact with the inner surface of the actuator to measure the maximum pulling force of the actuator and the half cylinder represents the case where only a small portion of the object is in contact with the inner surface of the actuator. The triangular prism represents the case where only a single point is originally in contact with the actuator. The results show that the gripper is generally capable of a minimum pulling force of approximately 25 N using thicker SMA wires if a single point is in contact with the actuator, but that if the object is performing a fully adaptive power grasp then its pulling force increases up to 30 N. The pulling force produced by the actuator is also sustained over a large displacement where the pulling force is near its maximum even after a displacement of 40 mm, which is much larger than the previously surveyed SMA-based soft grippers. The gripper finally loses its grip on the jig after a displacement of 60 mm for either the elliptical of half circle jigs. In the case of the inverted triangular prism, the gripper transitions from a single point of contact on the inside of the fingers to a fingertip grasp at approximately 40 mm and loses its grasp at a displacement of 72 mm. The behavior of the gripper was the same regardless of the diameter of the SMA wires used, but the pulling force was significantly increased.

The elliptical prism and the inverted triangular prism tests were repeated with the actuator wrapped in a TPU coated polyester sleeve which allows for a very low friction coefficient between the actuator and the jig (Fig. 7i–l). Results show that the maximum pulling force of the actuator reduces to 7 N and 13 N for the elliptical prism for the different SMA wire diameters. In the case of the inverted triangular prism, the actuator has the same maximum pulling force, but the force generated after the grip changes to the inside of the finger is significantly weakened especially for the thicker SMA wires. These results show that the actuator is capable of a reasonable pulling force for objects of moderate weights, but that friction may be needed to lift heavier objects.

**Figure 8:**
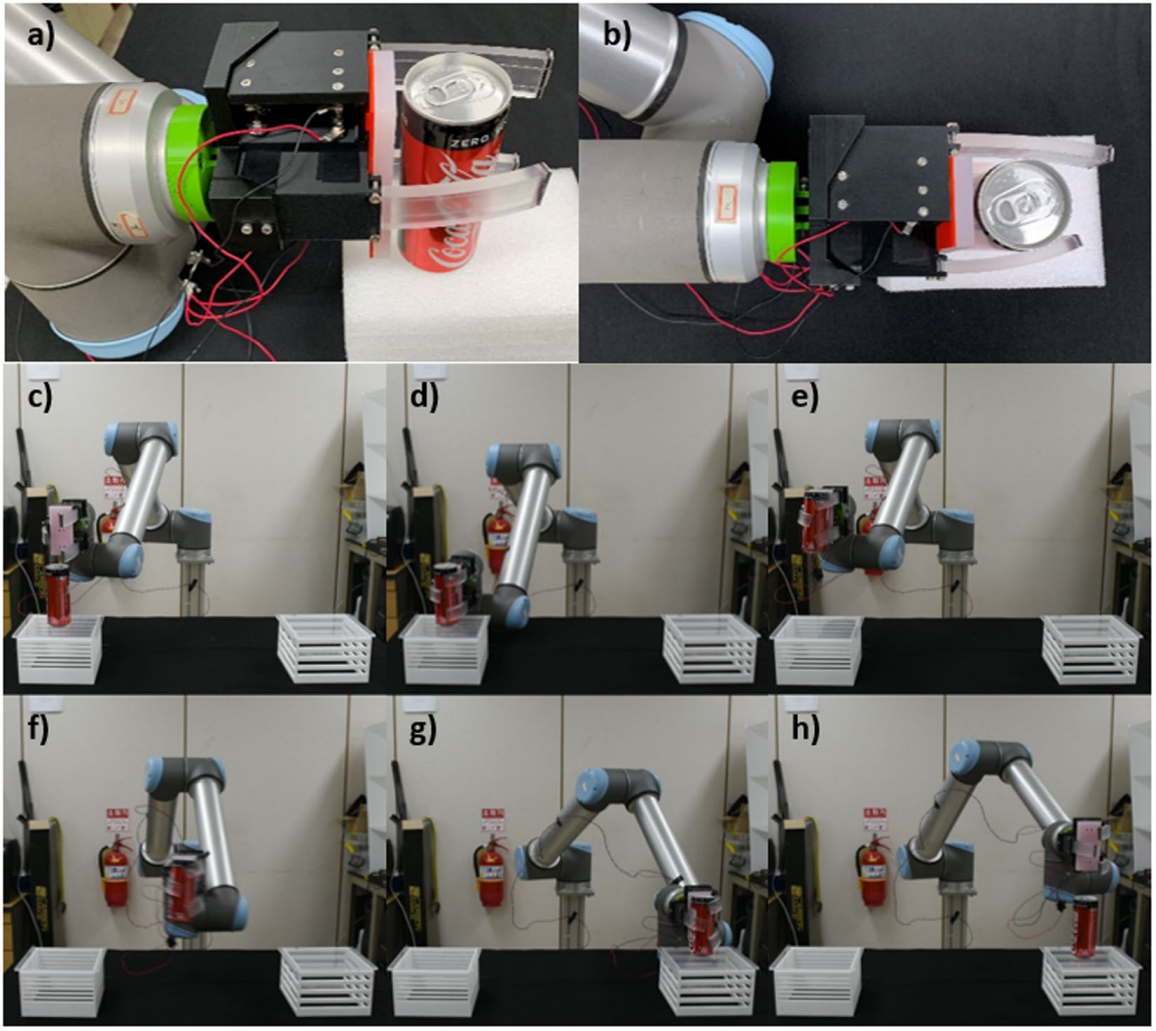
(**a**–**b**) Gripper installed on a robotic arm, and the gripper (**c**,**d**) grasping, (**e**–**g**) moving, and (**h**) releasing a can of soft drink.

The gripper was then installed as the end-effector of a robotic arm (UR10, Universal Robots) (Fig. 8a,b). A can of soft drink with a weight of 273 g was successfully grasped, moved, and released by the gripper installed on the robotic arm (Fig. 8c–h). This experiment demonstrates that the proposed modular gripper can be easily implemented as part of a robotic arm despite the use of extremely long SMA wire tendons.

## Discussion

The proposed SMA-based soft actuator uses SMA wires as free-sliding tendons to overcome the limitations of previous SMA-based soft actuator designs. SMA wire-based soft actuators had the SMA wire embedded into the polymeric matrix in contact with the matrix throughout such that the active length of the SMA wire is limited to the length of the matrix. As SMA wires have a limited actuation strain, SMA-based soft actuators were heavily limited by this. Different designs had been proposed to produce large deformations despite this limitation, but generally at the detriment of the force of the actuator. Using long free-sliding SMA wires allows to use very long SMA wires that produce much larger linear deformations and to use the deformation to produce large deformations without using design solutions that reduce the force of the actuator. Previous work has used externally located SMA springs to enable the use of springs, but this required thicker wires in the fabrication of the springs that required more power input for actuation. Furthermore, longer springs resulted in unwieldy system sizes. The use of SMA wires as the tendon itself enables the use of already established techniques for building compact actuator packages externally to the polymeric structure through pulleys^12^.

The developed gripper was able to grasp heavier objects than previous SMA-based grippers due to producing larger deformations and larger bending forces. This is directly due to the use of SMA wires much longer than the polymeric matrix through their use as a tendon and the possibility to position them at any eccentricity within the matrix since their stroke can be adjusted by increasing their length. The use of bearings to place the leading SMA wire into a compact package also enabled the actuators to be easy to use and assembled in any desired configuration to form a capable gripper that can be mounted on a robotic arm. The module could easily be designed in a more compact manner using more rigid material, smaller bearing and a better tendon arrangement.

Within the surveyed literature, the largest pulling force for an SMA wire-based soft gripper was 1.4 N without a stiffening mechanism and 6 N using fusible alloy as a stiffening structure while the developed soft robotic gripper produced a maximum pulling force of 25 N. It is possible to further increase the bending force of the actuator by increasing the number of SMA wires in parallel within the matrix, by increasing the diameter of the SMA wires, and by further increasing the length of the tendons. This would, however, increase the energy requirements of the gripper and might require the use of active cooling of the tendons to prevent excessive heat accumulation within the matrix and within the module housing the SMA wires. Other heating methods than Joule heating may be necessary to increase the heating speed and energy efficiency of the actuator if extremely long lengths of SMA wire are used for a single actuator. An alternative explored in other previous SMA-related works that could also be used in the developed gripper is to optimize the design of the matrix to improve the performance of the actuator. This solution is not exclusive with and could be used in tandem with long SMA tendons to further improve the performance of this type of soft actuator.

Pneumatic soft grippers are currently the most commonly used type of soft gripper using mechanical forces to grasp objects due to their large deformations and large force. However, their force diminishes rapidly throughout the motion as significant work is required to deform and expand the polymeric matrix. The force produced by the actuator presented in this paper decreases steadily but slower than polymer-based soft pneumatic grippers such that their performance for grasping could rival or exceed previously developed soft pneumatic grippers while being lighter, significantly quieter and not requiring an external pneumatic source. Further design improvements will be required before SMA-based soft grippers can truly compete with other types of grippers, but the present work.

## Conclusion

An important design limitation of previously developed SMA-based soft actuators was that the active length of the SMA wire is equal to that of the polymeric matrix which limits the available strain and constrains the performance of the actuator. In this work, the use of SMA wires as free-sliding tendons within the matrix and fixed externally allows to decouple the length of the matrix and that of the SMA wires such that it is possible to use much longer SMA wires than the polymeric matrix. This was used to realize large increases in performance without altering the design of the matrix with the largest achieved bending angle reaching 400° achieved, the largest reported for SMA-based soft bending actuators, and a tip force of 0.89 N. This design could also be usable for other types of wire-like smart materials coupled with a polymeric matrix whose finite strain limits their performance. The second major advantage of this concept is that the SMA wire can be packaged into a module using pulleys as done previously for non-soft mechanical devices while maintaining a compact form factor for the overall soft actuation system^12^.

This concept was then applied to a modular soft gripper where each module contains an actuator driven by long SMA wire tendons packaged compactly using bearings. The soft gripper was demonstrated to be able to grab a variety of objects shapes and weight in different orientations, and even of producing pulling forces up to 30 N. It was then installed as the end-effector of a robotic arm and demonstrated its capability to grab and move objects in both the power grasp and the fingertip grasp.

The proposed tendon design can significantly improve the performance of SMA-based soft actuators and even begin to make them compete with soft pneumatic actuators while maintaining the low weight, noiselessness, low cost and simple actuation method of SMA-based soft actuator. This design could be adapted to produce capable robots, mechanisms and medical tools. Future work will concentrate on further improvements of the gripper to produce faster actuation and stronger grasping forces for industrial uses.

## Data Availability

All data generated or analyzed during this study are included in this published article.
